# Identification of folate receptor α (FRα) binding oligopeptides and their evaluation for targeted virotherapy applications

**DOI:** 10.1038/s41417-019-0156-0

**Published:** 2020-01-06

**Authors:** Sarah L. Hulin-Curtis, James A. Davies, Davor Nestić, Emily A. Bates, Alexander T. Baker, Tabitha G. Cunliffe, Dragomira Majhen, John D. Chester, Alan L. Parker

**Affiliations:** 1 Division of Cancer and Genetics, School of Medicine, Cardiff University, Heath Park, Cardiff, CF14 4XN UK; 2 Division of Molecular Biology, Ruđer Bošković Institute, Bijenička cesta 54, 10000 Zagreb, Croatia; 3 Velindre Cancer Centre, Whitchurch, Cardiff CF14 2TL UK

**Keywords:** Genetic vectors, Genetic engineering

## Abstract

Oncolytic virotherapies (OV) based on human adenoviral (HAdV) vectors hold significant promise for the treatment of advanced ovarian cancers where local, intraperitoneal delivery to tumour metastases is feasible, bypassing many complexities associated with intravascular delivery. The efficacy of HAdV-C5-based OV is hampered by a lack of tumour selectivity, where the primary receptor, hCAR, is commonly downregulated during malignant transformation. Conversely, folate receptor alpha (FRα) is highly expressed on ovarian cancer cells, providing a compelling target for tumour selective delivery of virotherapies. Here, we identify high-affinity FRα-binding oligopeptides for genetic incorporation into HAdV-C5 vectors. Biopanning identified a 12-mer linear peptide, DWSSWVYRDPQT, and two 7-mer cysteine-constrained peptides, CIGNSNTLC and CTVRTSAEC that bound FRα in the context of the phage particle. Synthesised lead peptide, CTVRTSAEC, bound specifically to FRα and could be competitively inhibited with folic acid. To assess the capacity of the elucidated FRα-binding oligopeptides to target OV to FRα, we genetically incorporated the peptides into the HAdV-C5 fiber-knob HI loop including in vectors genetically ablated for hCAR interactions. Unfortunately, the recombinant vectors failed to efficiently target transduction via FRα due to defective intracellular trafficking following entry via FRα, indicating that whilst the peptides identified may have potential for applications for targeted drug delivery, they require additional refinement for targeted virotherapy applications.

## Introduction

Ovarian cancer is the sixth most common cause of cancer death in the UK, resulting in over 4000 deaths each year in the UK and 152,000 deaths worldwide (https://www.cancerresearchuk.org/health-professional/cancer-statistics/statistics-by-cancer-type/ovarian-cancer). Approximately 90% of ovarian cancers are of epithelial origin, categorised as serous, mucinous or endometroid [1]. The disease is typically asymptomatic in the early stages, commonly leading to late patient presentation and diagnosis and a 5-year survival rate of 46% [2]. Platinum-based chemotherapies are the first-line treatment; however, patients often develop chemo-resistance (https://www.cancerresearchuk.org/health-professional/cancer-statistics/statistics-by-cancer-type/ovarian-cancer) [2].

The use of adenoviral-based oncolytic virotherapies holds significant promise as anti-cancer agents due to their ability to self-amplify within tumours, lyse cells inducing immunogenic cell death, and express therapeutic modalities encoded within the viral genome. In the context of ovarian cancer, their deployment clinically is especially appealing since local delivery to tumour metastases via the intraperitoneal route is feasible, bypassing many restrictions that are associated with delivery via the bloodstream. To date the efficacy of HAdV-C5, the most commonly used HAdV vector for cancer gene therapy, has been hampered due to a lack of selectivity for cancer cells [3–5]. One approach is to identify ligands for cellular receptors that are unique to or overexpressed on tumour cells with low or absent expression in normal tissue. Cancer-targeting peptides have the potential for targeted drug delivery, enhancing therapeutic benefit and minimising off-target toxicities [6]. For virotherapy applications, incorporation of cancer-targeting peptides into the HI loop of the adenoviral fibre knob, the capsid protein that interacts with the native adenoviral receptor coxsackie adenovirus receptor (CAR) for cell entry, is an opportune targeting strategy for developing high-affinity, retargeted HAdV vectors [7]. Recently, we described such an approach to develop a “precision virotherapy” based on targeting the tumour associated integrin, αvβ6 using a previously identified, 20-amino-acid αvβ6-binding peptide, A20. The resultant virotherapy, Ad5_NULL_-A20, demonstrated a remarkable ability to purge xenografted SKOV3 ovarian cancers in an intraperitoneal model of ovarian cancer [8]. However, αvβ6 is overexpressed in around 33% of advanced ovarian cancers [9], and therefore the majority of patients presenting with late-stage ovarian cancer may not be amenable to precision virotherapies targeted to αvβ6.

Retargeting HAdV vector tropism towards the folate receptor alpha (FRα) represents an excellent approach for treating ovarian cancer. The FRα is a glycosylphosphatidylinositol (GPI) anchored membrane protein that binds and internalises folate for purine and thymidine DNA biosynthesis, repair and methylation. The FRα confers a tumour growth advantage and is positively associated with histological stage and grade and poor overall survival. In normal tissues, the expression of FRα is strictly confined to the apical membrane of polarised epithelial cells and is not usually expressed in normal ovarian epithelium but expressed in over 70% of primary and 80% of recurrent ovarian cancers [10]. The folate receptor represents an attractive target for the development of folate receptor nanoparticles for cancer detection and treatment. Such folate nanoconjugates consisting of polymers, micelles, dendrimers and liposomes, to name but a few, show promise in the approach of nanoparticle targeting of folate receptor-positive cancer cells and is reviewed elsewhere [11]. In the context of viral retargeting to the folate receptor, a previous study showed that chemical conjugation of folate to a murine Moloney leukaemia (retrovirus) vector failed to transduce folate receptor-positive cells. Although the folate-derivatised virus showed significant and specific binding to cells, the virus was not efficiently internalised, probably due to poor endocytosis of virus by folate receptors, perhaps incompatible with the native tropism of the virus. This suggests that even tight binding of virus to the cellular membrane does not always translate to efficient transduction capabilities [12]. Indeed, there is controversy as to whether the folate receptor pathway is a viable route for nanoparticles and viral vectors. Engineering adenoviral vectors retargeted to the folate receptor represents an excellent approach to addressing this question.

Phage display is a selection procedure for elucidating peptides binding partners to a given target of interest [13–15]. Commercial phage libraries are commonly based on the filamentous bacteriophage M13, typically consisting of 10^10^ randomised peptide sequences expressed as fusions to the bacteriophage coat protein pIII. Biopanning against whole cells requires incubating phage library with cells expressing the target of interest, washing away unbound phage before specifically eluting phage bound to the receptor of interest by competitive inhibition using a known receptor interacting agonist. Eluted phage is then collected, amplified and enriched for target-specific bound phage in subsequent rounds of bioselection.

The DNA encoding each peptide within the virion is physically linked to the peptide expressed on the coat protein allowing rapid elucidation of the DNA sequence of target-bound peptides. Although a number of pre-clinical phage display studies show promise for the utility of cancer-targeting peptides [16, 17], clinical data demonstrate the need for further optimisation for translational success in terms of improving receptor affinity, enzyme instability and pharmacokinetics [18, 19]. A number of clinical trials are exploring the potential therapeutic opportunities for targeting the FRα and are reviewed extensively elsewhere [20]. The monoclonal FRα antibody Farletuzumab (MORAb-003) shows conflicting efficacy data, whilst limited efficacy was demonstrated in trials evaluating the folate-conjugated drug Vintafolide (MK-8109, EC145). IMGN853 (Mirvetuximab soravtansine), an anti-FRα antibody conjugated to a cytotoxic drug is currently undergoing Phase I clinical trials [21]. In contrast to antibody-based agents, small cancer-targeting peptides have the advantage of their small size, potentially permitting easy penetration into tissue, reduced immunogenicity and easy synthesis and scale-up [18]. Peptides identified from phage libraries that have been used for HAdV retargeting to the tumour vasculature include the RGD-binding cellular integrins [22], NGR peptide-binding aminopeptidease N (APN) [23] and human epidermal growth factor (EGFR) GE11 peptide [24]. HAdV-based virotherapies can be readily genetically modified to incorporate peptides targeting cancer-restricted receptors within permissive regions of the adenoviral fibre knob protein; however, further refinements are often necessary to overcome limitations in clinical efficacy associated with off-target uptake [25].

In the present study, we performed whole-cell biopanning of SKOV3 ovarian carcinoma cells using phage display libraries to identify FRα-specific peptides and tested their binding specificity in vitro. To evaluate the potential to generate a “precision virotherapy” targeted to FRα, we genetically incorporated FRα-binding oligopeptides into the HI loop of the adenoviral fibre knob domain and evaluated their ability to direct viral tropism towards FRα in vitro.

## Materials and methods

### Cell lines

SKOV3 cells (human ovarian adenocarcinoma derived from ascites) were originally obtained from the American Type Culture Collection (ATCC). Cells were cultured and maintained in Dulbecco's Modified Eagle's medium (DMEM) supplemented with 10% foetal calf serum (FCS), 2 mM l-glutamine, 100 U/mL penicillin and 100 µg/mL streptomycin. Cells were maintained at 37 °C and 5% CO_2_. All reagents were purchased from Gibco or Thermo Scientific (Paisley, UK). Cells were routinely tested for mycoplasma (every 3–6 months).

### Flow cytometric analysis of receptors expressed on SKOV3 cells

SKOV3 cells were seeded at a density of 1.5 × 10^5^ cells per well in a 96-well plate. Cells were washed in 200 µL of wash buffer (PBS/1% BSA) and incubated with 100 µL of wash buffer containing 1:500 of mouse anti-human monoclonal antibody against CAR (RmcB, Millipore, Watford, UK), 1:50 of mouse anti-human monoclonal anti-folate binding protein antibody (clone EPR4708(2)) (Abcam, UK) or mouse IgG control antibody (Santa Cruz Biotechnology, Heidelberg, Germany) for 1 h on ice. Cells were washed three times and incubated with a 1:500 dilution of goat anti-mouse Alexa Fluor 647 antibody (Invitrogen, UK) for 30 min on ice. Cells were fixed in 4% paraformaldehyde for 20 min at 4 °C. A total of 2 × 10^4^ gated events were acquired in channel FL-4 on a BD Accuri C6 (BD Biosciences, USA) flow cytometer and data analysed in BD Accuri C6 software version 1.0.264.21 (Becton Dickinson, USA). CAR and FR binding was analysed by flow cytometry relative to IgG isotype binding.

### Phage libraries

The Ph.D.−12, Ph.D.−7 and Ph.D.−C7C Phage Display Peptide Libraries were purchased from New England Biolabs (UK). The combinatorial library of random dodecapeptides (Ph.D.−12) and heptapeptides (Ph.D.−7 and Ph.D.−C7C) are fused to a minor coat protein (pIII) of M13 phage. The displayed peptides (12-mer and 7-mer, respectively) are expressed at the N-terminus of pIII. The library consists of 1 × 10^13^ plaque forming units (pfu)/mL phage with 10^9^ random sequences yielding ~100 copies of each sequence.

### Phage biopanning

SKOV3 cells were seeded at 5 × 10^5^ cells per well in a 6-well plate in DMEM, 10% FCS and cultured at 37 °C, 5% CO_2_. After 24 h, cells were cooled to 4 °C for 30 min. Cells were washed twice with cold PBS. Phage library was added at 2 × 10^11^ phage particles in a total volume of 1 mL DMEM/1% BSA and incubated with gentle rocking at 4 °C for 1 h. Unbound phage was removed (and stored at 4 °C). Cells were washed four times for 2 min in PBS/1% BSA. Bound phages were eluted by addition of 1 mL of folic acid (Acros organics, Fisher Scientific, UK) in PBS (100 µg/mL) by competitive inhibition for 1 h at 4 °C. Phages were stored at 4 °C until analysis (unamplified eluate). Unamplified eluted phage was titred to determine the success of each round of biopanning.

### Phage titering

A single ER2738 bacterial colony was cultured in Luria Bertani (LB) broth overnight with tetracycline at 37 °C, 150 rpm. Tenfold serial dilutions of unamplified phage (10^1^–10^4^) in LB were prepared overnight and added to overnight ER2738 cultures. After immediate and brief vortexing, diluted phage was incubated at room temperature for 1–5 min and transferred to a tube containing 3 mL of 4 °C agarose top. After a brief vortex, bacterial cultures were poured onto pre-warmed LB/IPTG/Xgal plates, cooled for 5 min and incubated overnight at 37 °C. The next day, plaques were counted to calculate number of plaque forming units.

### Phage amplification

A single ER2738 bacterial colony was cultured in LB broth overnight with tetracycline at 37 °C, 150 rpm; 3 mL of overnight culture was added to 20 mL of LB and unamplified eluate added. Phages were amplified at 37 °C, 150 rpm. The culture was centrifuged at 10,000 rpm (12,000×*g*) for 20 min at 4 °C. The supernatant was transferred to a fresh tube and centrifuged. The upper 80% (~19 mL) of the supernatant was transferred to a fresh tube and 1/6th volume (~3 mL) of PEG/NaCl added. Phages were precipitated at 4 °C overnight.

### PEG precipitation

The PEG precipitate was centrifuged at 10,000 rpm for 15 min at 4 °C. The supernatant was discarded and re-centrifuged briefly to remove residual supernatant. The phage pellet was resuspended in 1 mL of TBS and centrifuged in a clean tube at 13,000 rpm, for 5 min at 4 °C. The supernatant was transferred to a clean tube, precipitated with 1/6th volume (~166 µL) of PEG/NaCl, incubated on ice for 30 min followed by centrifugation at 13,000 rpm for 10 min at 4 °C. The supernatant was discarded and centrifuged briefly to remove residual supernatant. The pellet was resuspended in 200 µL of TBS, 0.025 NaN_3_ and centrifuged for 1 min to pellet residual insoluble material. The supernatant was transferred to a clean tube as the amplified eluate. The amplified eluate was titred as described above using 10^8^–10^11^ dilutions of amplified phage eluate.

### Plaque amplification and purification

Unamplified eluate was titred as described above. Plates were incubated at 37 °C for no longer than 18 h. Plaques (blue) were picked using a sterile pipette tip and incubated for 2 h at room temperature. Using overnight ER2738 cultures in LB, a 1:100 dilution (30 µL) was added to 3 mL of LB. Cultures were incubated at 37 °C overnight (without shaking). A total of 1.5 mL of culture was analysed for sequencing by centrifugation at 14,000 rpm for 10 min; 500 µL of phage-containing supernatant was transferred to a fresh tube, the remaining 1 mL re-centrifuged and the upper 80% of the supernatant transferred to a fresh tube (amplified phage stock). A total of 500 µL of phage-containing supernatant was added to 200 µL of PEG/NaCl, inverted to mix and incubated for 10 min at room temperature. Phage was centrifuged at 14,000 rpm for 10 min at 4 °C. The supernatant was discarded and pellet resuspended in 100 µL of iodide buffer with vigorous tapping of the tube; 250 µL of ethanol was added, incubated for 10 min at room temperature and centrifuged at 14,000 rpm for 10 min at 4 °C. The supernatant was discarded and the pellet washed with 70% ethanol, dried briefly at 37 °C and pellet resuspended in Tris/EDTA. A total of 5 µL was used for direct sequencing.

### Peptide synthesis

TVRTSAE incorporating a thiol group (TVRTSAEGGCGG) was synthesised by ArchieChem (UK) and conjugated to Alexa Fluor 647 C2 maleimide (Thermo Fisher, UK). TVRTSAE labelled with FITC (TVRTSAEGGCGG-COOH) was commercially synthesised by Innovagen (Lund, Sweden), converted to acetate salt for use on live cells. Peptide purity and mass was determined by HPLC and ESI-MS, respectively.

### Phage cell-binding assay

SKOV3 cells were seeded in 8-well Nunc Lab-Tek^TM^ II chamber slides (Thermo-Scientific, UK) at a density of 2 × 10^4^ cells/well in 300 µL of DMEM supplemented with 10% FCS, 2 mM l-glutamine, 100 U/mL penicillin and 100 µg/mL streptomycin. Cells were incubated at 5% CO_2_, 37 °C and grown to ~80% confluency (24 h). Cells were washed twice with PBS, infected with 2 × 10^10^ phages in PBS/1% BSA in a total volume of 100 µL, diluted as appropriate by titering overnight cultures on LB/IPTG/Xgal plates. Cells were incubated for 2 h at 4 °C. Cells were washed five times in cold PBS/1%BSA/0.1% Tween-20 and incubated with mouse anti-M13 antibody (Abcam, ab9225) at a 1:50 dilution in PBS/1% BSA for 1 h at 4 °C. Bound phage were detected by incubating cells with Alexa Fluor 488 goat anti-mouse (Invitrogen, A11017) at a dilution of 1:2000 in PBS/1% BSA for 45 min at room temperature. Cells were washed four times in cold PBS/1% BSA and fixed with 4% PFA for 20 min at room temperature. Cells were washed with PBS and slides mounted with Prolong Diamond with DAPI (Invitrogen).

### Flow cytometry analysis of TVRTSAE peptide dose-response and cell binding

SKOV3 were seeded at a density of 1 × 10^5^ cells per well in a 96-well plate. Cells were washed in 200 µL of wash buffer (PBS/1% BSA) and incubated with 100 µL of wash buffer containing either 100 µM, 300 µM or 500 µM TVRTSAEGGCGG peptide or PBS for 1 h at 4 °C. Cells were washed and incubated with wash buffer for 1 h at 4 °C. Cells were fixed in 4% paraformaldehyde for 20 min at 4 °C. A total of 2 × 10^4^ gated events were acquired in channel FL-4 on a BD Accuri C6 (BD Biosciences, USA) flow cytometer and data analysed in BD Accuri C6 software version 1.0.264.21 (Becton Dickinson, USA). TVRTSAEGGCGG peptide binding was analysed by flow cytometry relative to PBS control.

### Immunofluorescence analysis of TVRTSAE peptide binding to folic-acid-treated cells

SKOV3 cells were seeded in 8-well Nunc Lab-Tek^TM^ II chamber slides (Thermo-Scientific, UK) at a density of 2 × 10^4^ cells/well in 300 µL of DMEM supplemented with 10% FCS, 2 mM l-glutamine, 100 U/mL penicillin and 100 µg/mL streptomycin. Cells were incubated in 5% CO_2_, at 37 °C and grown to approximately 80% confluency (24 h). Cells were washed twice with PBS and incubated with either 0.5 mM folic acid in PBS/1% BSA or complete medium in a total volume of 300 µL/well for 1 h at 4 °C. Cells were washed with PBS and incubated with 0.5 mM FITC-labelled TVRTSAEGGCGG-COOH peptide in PBS/1% BSA in a total volume of 300 µL/well for 1 h at 4 °C. PBS was used as a no-peptide control. Cells were washed four times in cold PBS/1% BSA and fixed with 4% PFA for 20 min at room temperature. Cells were washed with PBS and slides mounted with Prolong Diamond with DAPI (Invitrogen).

### Predictive modelling of HAdV-C5 fibre knob protein containing FRα-binding peptide inserts

The structure of the HAdV-C5 fibre knob containing the peptide insert was simulated using SWISS-MODEL [26] and the best available crystal structure of the HAdV-C5 fibre knob protein as a template (PDB 6HCN) [27]. Structures were visualised in PyMol [28].

### Generation of HAdV vectors

A panel of recombinant HAdV genomes were produced using HAdV-C5 luciferase-expressing genomes rendered replication-deficient by deletion of early genes (ΔE1/ΔE3), with and without the KOI mutation that ablates binding of adenovirus to CAR, introduced by mutation of S408E and P409A in the AB loop. FRα-specific peptides DWSSWVYRDPQT, CIGNSNTLC and CTVRTSAEC were inserted in the HI loop of the fibre knob at amino acid position 542 and the KO1 mutation was introduced by S408E and P409A in the AB loop. Recombinant HAdV-C5 genomes were generated by homologous recombination in *Escherichia coli* strain SW102. Firstly, a SacB selection cassette was inserted in the HAdV-C5 genome and then replaced with each of the oligonucleotide sequences representing each FRα-specific peptide. DNA extraction and purification was performed by mini-preparation (Qiagen). Selection cassettes and olignonucleotides containing 100 bp homology arms were generated by PCR using Expand Hi-Fi PCR (Roche Applied Science, UK). Confirmation of correct clone sequences was confirmed by direct DNA sequencing using a commercial sequencing service.

For generating adenovirus, miniprep DNA was amplified in a 10-mL culture for ~8 h and added to a 250-mL culture overnight. Purification was performed using the BacMax 100 kit (Macherey-Nagel, Duren, Germany) and transfected into T-Rex-293 cells using the Effectene transfection kit (Qiagen) in T25 tissue culture flasks. When the cytopathic effect (CPE) was achieved, cells were pelleted and virus extracted using tetrachloroethylene (Fisher Scientific, Loughborough, UK). The initial virus stocks were amplified by infecting 5 × T150 confluent T-Rex-293 flasks. Cell pellets were extracted as before and the virus purified by centrifugation using two rounds of caesium chloride (CsCl) gradient. CsCl was removed from the virus by dialysing against buffer containing 10% glycerol, 10 mM Tris-HCl (pH 7.8), 135 mM NaCl, and 1 mM MgCl_2_·6H_2_O. Viral titres were determined using the micro bicinchoninic acid (BCA) assay (Pierce) with the assumption that 1 μg of protein equals 4 × 10^9^ viral particles (vp).

### Western blotting

The structural integrity of the HAdV-C5 fibre knob proteins incorporating peptide insertions was assessed by Western blotting. A total of 1 × 10^10^ vp/virus stock were run on pre-made 10% NuPAGE polyacrylamide gels (Invitrogen, Paisley, UK) by SDS-PAGE and transferred to Hybond-P nitrocellulose membrane (GE Healthcare Life Sciences, Little Chalfont, UK) by semidry blotting. Nitrocellulose membranes were treated with 5 mL of Pierce Miser antibody extender (Thermo Scientific) for 10 min and washed seven times with distilled water. Membranes were blocked in 5% milk in Tris-buffered saline containing 0.05% TWEEN-20 and 0.05% Triton X-100 (TBS-T) overnight at 4 °C. The membrane was incubated in primary anti-adenovirus fibre antibody 4D2 (Abcam, ab3233) (1:2000) at 37 °C for 1 h, washed five times for 5 min in TBS-T and incubated in anti-mouse IgG-HRP conjugate (1:2000; Insight Biotechnology Ltd., Wembley, UK) for 1 h at room temperature. After washing a further five times for 5 min in TBS-T, the membrane was incubated for a maximum of 10 min in Super Signal West Pico Chemiluminescent substrate (Thermo Scientific) and analysed on GelDoc autoChemi camera (Ultra-Violet Products Ltd., Cambridge, UK).

### In vitro adenovirus-mediated cell transduction

In brief, cells were seeded at a density of 2 × 10^4^ cells/well in a 96-well plate. After 24 h, cells were infected with luciferase-expressing HAdV vectors at a dose of 5000 vp/cell in a total volume of 100 µL of serum-free medium and incubated at 5% CO_2_, 37 °C for 3 h. The medium was removed and replaced with 200 μL of complete medium (RPMI 1640 medium supplemented with 10% (v/v) FCS, 100 U/mL penicillin, 100 µg/mL streptomycin) and cultured for an additional 45 h. Cells were lysed in 1X Cell Culture Lysis Buffer (Promega, UK) and frozen at −70 °C. The cells were thawed and 20 µL of cells were mixed with 100 µL of luciferase assay reagent in a white 96-well plate. Luciferase activity in relative light units (RLU) was measured immediately using a multimode plate reader (FLUOstar Omega, BMG Labtech, Aylesbury, UK). Samples were normalised for total protein content, as measured by BCA assay in RLU/mg protein. A total of 2 × 10^4^ gated events were acquired in channel FL-1 on a BD Accuri C6 using the plate reader as described above.

### Adenovirus labelling and confocal microscopy

Adenovirus particles were incubated with a 20-fold excess of Alexa Fluor 488-TFP (Molecular Probes) for 1.5 h at room temperature in 10% glycerol/100 nM NaHCO_3_/PBS buffer, pH 7.2. Unbound dye was removed from the labelled virus using two Zeba Spin desalting columns (Pierce) with exchange buffer 10% glycerol in PBS buffer.

SKOV3 cells were seeded in 24-well plates on coverslips at a density of 2 × 10^4^ cells/well. Two days later, labelled adenoviruses were added to the cells (250,000 vp/cell) in serum-free medium and incubated on ice for 45 min. Cells were then transferred to 37 °C and after 30 or 60 min were fixed (2% paraformaldehyde in PBS for 12 min at room temperature), permeabilised (0.1% Triton X-100 in PBS for 2 min at room temperature) and incubated with Alexa Fluor 555 Phalloidin (Thermo Fisher). Coverslips were mounted using Fluoromount G (Southern Biotech) containing DAPI for labelling the nuclei. Leica TCS SP8 microscope with 63× objective was used for imaging. The images were analysed using LAS X (Leica Microsystems, Germany) software and they are showing maximum projections of confocal stacks.

### Statistical analyses

Data are presented as experiments performed in triplicate or quadruplicate as indicated in the relevant figure legends. All analyses and graphs were created in GraphPad Prism version 6.03 (GraphPad Software Inc., La Jolla, CA, USA). *P* values ≤ 0.05 were considered statistically significant.

## Results

### Identification of FRα-binding peptides

**Fig. 1:**
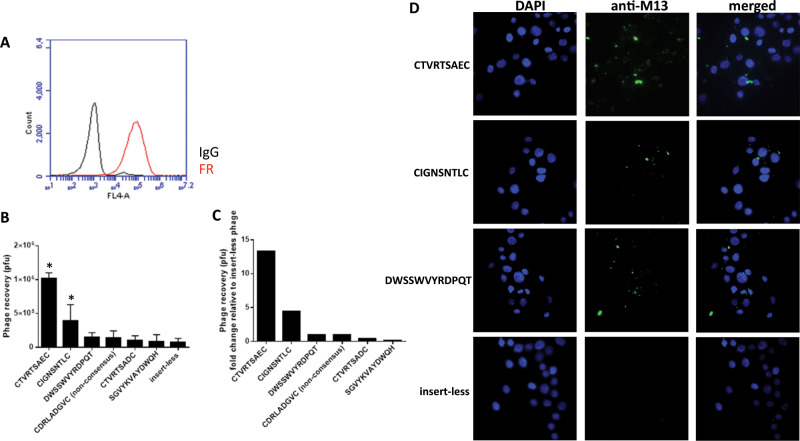
Identification and characterisation of FRα binding oligopeptides from phage display libraries. **a** Characterisation of FRα expression on SKOV3 cells. **b** Phage peptide recovery from SKOV3 cells eluted with folic acid. Cell binding of homogeneous selected phage clones recovered from SKOV3 cells eluted with folic acid, **c** fold change relative to insert-less phage. Bars represent the mean ± S.D. #*P* ≤ 0.05 versus DRLADGV (non-consensus) control peptide. *P ≤ 0.05 versus insert-less control peptide. **d** Phage clones bind SKOV3 cells in vitro. 7-mer cyclic phage peptides (CTVRTSAEC, CIGNSNTLC) and 12-mer linear peptides (DWSSWVYRDPQT) bind to SKOV3 cells. Cells were infected with 2 × 10^10^ pfu of phages at 4 °C and detected by anti-M13 antibody. An empty (insert-less) phage clone was used as a control.

FRα expression on SKOV3 cells was first confirmed by flow cytometry (Fig. 1a) to ensure this was an appropriate cell line for our biopanning strategy. The Ph.D.−7 (7-mer), Ph.D.−C7C (7-mer) and Ph.D.−12 (12-mer) phage-display libraries displaying random peptides on the coat protein were incubated on SKOV3 cells and eluted with folic acid (100 µg/mL) to elute FRα-specific binding peptides for each library. A total of six rounds of biopanning was performed. Phage clones were randomly picked and characterised by DNA sequencing. Consensus sequences iterated for the 12-mer and 7-mer (Ph.D.−C7C) libraries are shown in Table 1. The linear 7-mer library (Ph.D.−7) failed to identify any consensus sequence. After each panning round, phages were titred to determine phage recovery. Homogenous solutions of phage-presenting peptides of interest were used in binding studies on SKOV3 cells (Fig. 1b) and fold-change relative to insert-less phage was calculated (Fig. 1c). CTVRTSAEC phage showed the highest fold increase in recovery on SKOV3 cells followed by CIGNSNTLC in comparison to the other phage clones. To further evaluate the binding of phage clones expressing the lead peptide sequences CTVRTSAEC, CIGNSNTLC and DWSSWVYRDPQT to SKOV3 cells in vitro, homogenous phage solutions were incubated on SKOV3 cells, and their binding detected using and anti-M13 antibody. Immunocytochemical analysis demonstrated that phage displaying CIGNSNTLC, DWSSWVYRDPQT and, in particular, CTVRTSAEC were better able to bind to SKOV3 cells compared to control phage (Fig. 1d).

**Table 1:** Frequency of phage peptide sequences derived from SKOV3 cell biopanning using the Ph.D.−12 and Ph.D.−C7C phage-display library.

Phage library	Frequency	Frequency (%)	Amino acid sequence
12-mer	6	10	DWSSWVYRDPQT
C7C	5	9	CIGNSNTLC
C7C	4	7	CTVRTSADC
12-mer	3	5	SGVYKVAYDWQH
C7C	2	4	CTVRTSAEC

### Evaluation of TVRTSAE-binding SKOV3 cells

**Fig. 2:**
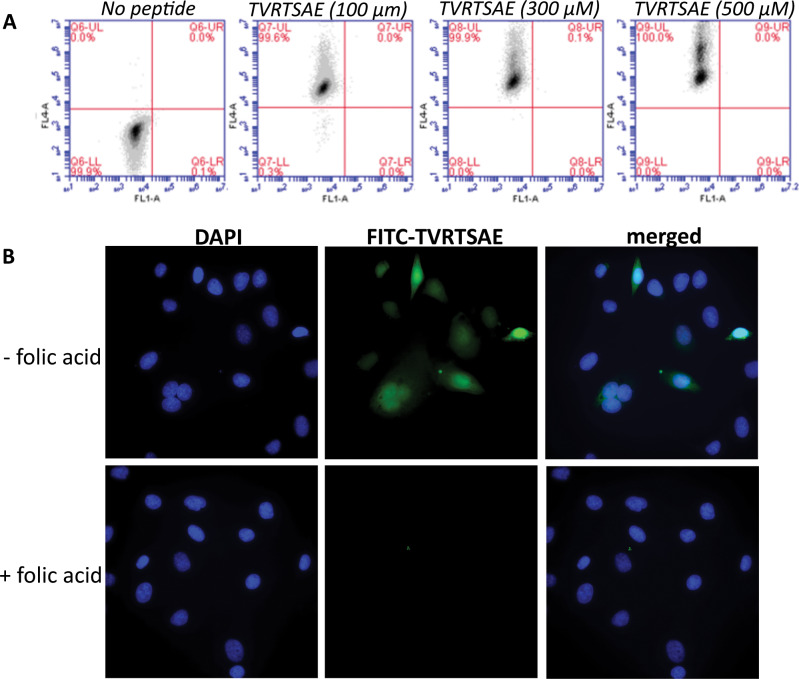
Evaluation and competitive inhibition of TVRTSAE peptide binding to FRα on SKOV3 cells. **a** Synthesised TVRTSAE peptide binding to SKOV3 cells increases with escalating peptide concentration. SKOV3 cells were incubated with increasing peptides concentration (100, 300 or 500 µM) for 1 h at 4 °C and analysed by flow cytometry. PBS was used as a no-peptide control. **b** FITC-TVRTSAE peptide binds to SKOV3 cells via the FRα. Representative immunofluorescence images show FITC-TVRTSAE peptide binding to SKOV3 cells. FITC-labelled TVRTSAEGGCGG-COOH peptide (0.5 mM) was incubated on SKOV3 cells in PBS/1% BSA for 1 h at 4 °C (upper panel). Cells treated with folic acid (0.5 mM) in PBS/1% BSA were incubated for 1 h at 4 °C prior to treatment with peptide (lower panel).

To determine whether the TVRTSAE peptide binds SKOV3 cells outside of the constraints of a phage virion, Alexa Fluor 647-labelled peptide (TVRTSAEGGCGG) was incubated at escalating doses of peptide; 100 µM, 300 µM and 500 µM and cell binding determined by flow cytometry (Fig. 2a). The data show a corresponding increase in SKOV3 cell binding with increasing peptide dose. No peptide (PBS) was used as a control. To confirm that TVRTSAE peptide binds SKOV3 cells via the FRα, FITC-conjugated peptide (TVRTSAEGGCGG-COOH) (500 µM) was tested for its ability to bind SKOV3 cells by competitive inhibition with pre-treatment of cells with folic acid (0.45 µM), the native ligand for FRα. Peptide binding was prevented when cells were pre-treated with folic acid as determined by immunocytochemistry (Fig. 2b).

### Predictive structural modelling of HAdV-C5 fibre knob protein containing FRα-binding oligopeptides

**Fig. 3:**
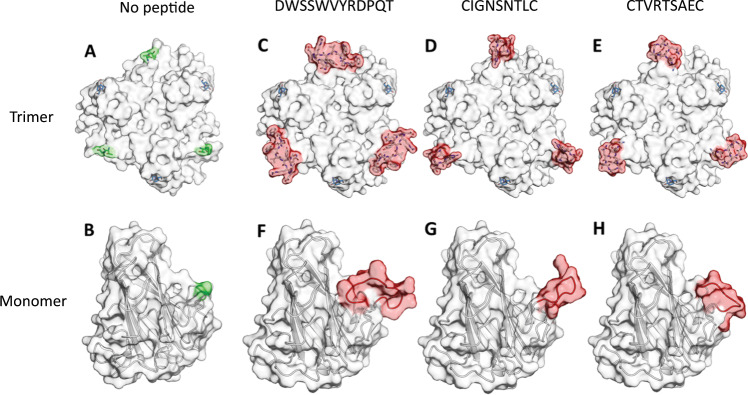
Predictive modelling of HAdV-C5 fibre knob proteins containing FRα-binding oligopeptides. Mutated residues involved in CAR binding (KO1 mutation, 408S, 409P, blue sticks) are distant from the insert site (green surface) in the adenovirus 5 fibre knob trimer (**a**) residing in the lateral portion of the monomeric unit (**b**). The trimers containing peptides (white sticks, red surface) DWSSWVYRDPQT (**c**), CIGNSNTLC (**d**) and CTVRTSAEC (**e**) are highlighted (red) within the trimeric fibre knob protein. In the monomeric unit of the fibre knob, peptides (red cartoon) DWSSWVYRDPQT (**f**), CIGNSNTLC (**g**) and CTVRTSAEC (**h**) are visualised and appear to possess different confirmations.

To assess the possibility that genetic incorporation of FRα-binding oligopeptides into the HAdV-C5 fibre knob protein might cause significant structural alterations to the protein, we performed modelling analysis of the predicted structures (Fig. 3). The structure of the HAdV-C5 knob protein (based on PDB 6HCN) containing each peptide insert was simulated using SWISS-MODEL, and the resultant structures visualised using PyMol. Each of the structures was modelled within the wild-type HAdV-C5 fibre knob protein as well in a CAR-binding ablating mutant, containing two mutations, S408E and P409A, within the fibre knob AB loop. The resultant structures demonstrated that the peptide insert was presented in a distal manner, extending away from the fibre knob protein, in a manner that ought to be favourable for receptor interactions. Peptide insertions were shown to be distant from the putative CAR-binding site within the fibre knob protein. The predicted structures therefore gave confidence that the resultant vectors should be viable and present FRα-interacting oligopeptides in a manner compatible with receptor engagement.

### Genetic engineering of HAdV-C5 vectors to incorporate FRα-specific peptides within the fibre knob protein

**Fig. 4:**
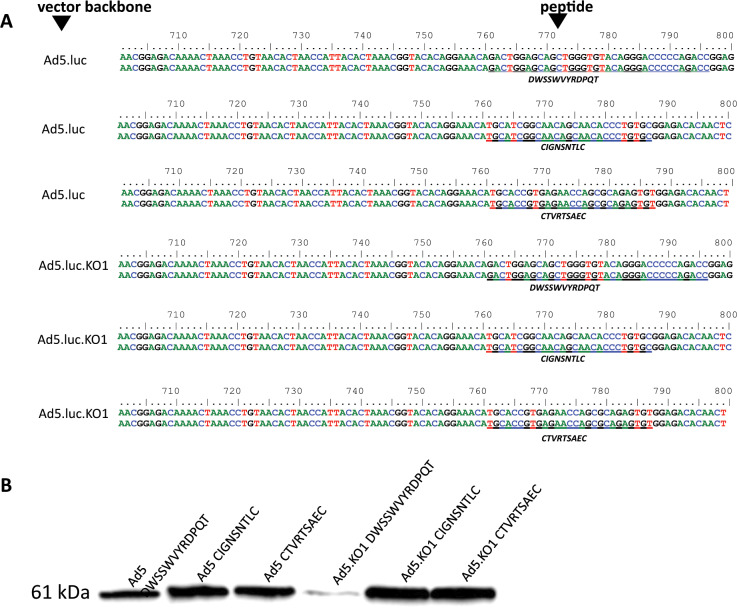
Production and validation of recombinant HAdV-C5 vectors containing FRα-binding oligopeptides within the fibre knob protein. **a** Direct sequencing of recombinant HAdV-C5 and HAdV-C5.KO1 vectors generated, confirming successful peptide integration. Translated nucleotide to amino acid sequences are shown in italics. **b** Representative western blot confirming the structural integrity of recombinant Ad fibre knobs using a mouse monoclonal antibody [4D2] specific to adenovirus fibre monomer and trimer. For simplicity, HAdV-C5 is abbreviated to Ad5 in the figure.

We developed a panel of retargeted HAdV vectors presenting FRα peptide-binding sequences in the adenoviral fibre knob as a retargeting approach for selective targeting to FRα (Table 2). Retargeted HAdVs were produced by AdZ homologous recombineering as described previously [29]. The HAdV-C5 parental vector was rendered replication-deficient by deletion of E1 and E3 genes (∆E1/∆E3). Peptide sequences were incorporated into the HAdV-C5 genome within the HI loop of the HAdV-C5 fibre knob domain after Thr541 (Table 2) since this region is permissible for incorporation of peptides [30]. CAR-binding ablated HAdV-C5 vectors were developed by incorporation of point mutations S408E and P409A (KO1) within the AB loop of the fibre knob domain (Table 2). Such KO1 mutations preclude interactions between adenovirus and the native receptor for cell entry CAR [31]. Direct sequencing of HAdV genomes within the region of homologous recombination and flanking regions confirmed correct adenoviral genomic sequences (Fig. 4a). The structural integrity of retargeted HAdV fibre knobs incorporating peptide insertions was evaluated by Western blot (Fig. 4b). All retargeted HAdV fibre knobs show a distinct band at 60 kDa, consistent with intact HAdV fibre knobs.

**Table 2:** Amino acid position of (peptide) DNA oligonucleotides inserted into the HI loop of the Ad5 and Ad5.KO1 fibre knobs by AdZ homologous recombineering.

	AB loop		HI loop			
Amino acid (fibre knob)	**408**	**409**	**540**	**541**		**543**
Ad5	S	P	E	T	- - - - - - - - - - - -	G
Ad5 DWSSWVYRDPQT	S	P	E	T	*DWSSWVYRDPQT*	G
Ad5 CIGNSNTLC	S	P	E	T	*CIGNSNTLC*	G
Ad5 CTVRTSAEC	S	P	E	T	*CTVRTSAEC*	G
Ad5.KO1	E	A	E	T	- - - - - - - - - - - -	G
Ad5.KO1 DWSSWVYRDPQT	E	A	E	T	*DWSSWVYRDPQT*	G
Ad5.KO1 CIGNSNTLC	E	A	E	T	*CIGNSNTLC*	G
Ad5.KO1 CTVRTSAEC	E	A	E	T	*CTVRTSAEC*	G

### Evaluation of FRα retargeted HAdV vector transduction

**Fig. 5:**
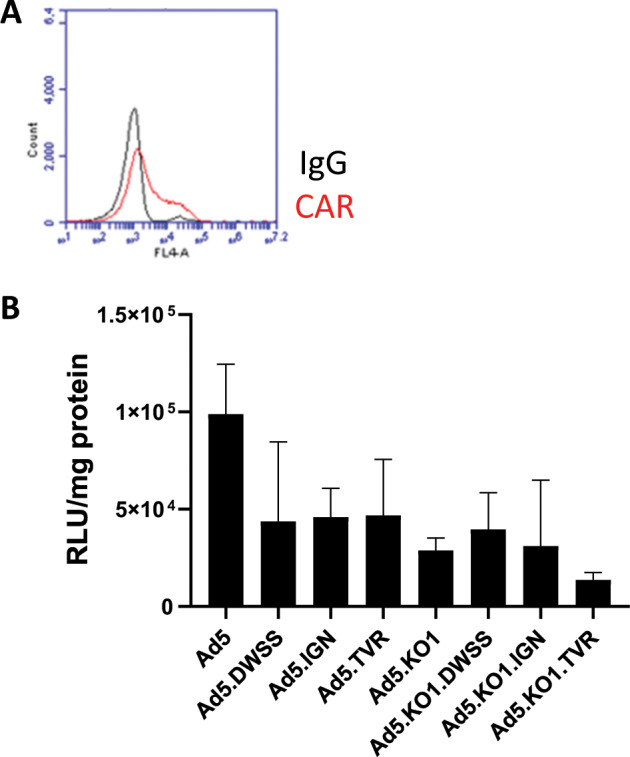
In vitro SKOV3 cell transduction of FRα retargeted HAdV-C5 incorporating peptide oligonucleotides in the HI loop of the fibre knob. **a** SKOV3 cell receptor expression of CAR as determined by flow cytometry. **b** SKOV3 cells were transduced with luciferase expressing HAdV-C5, HAdV-C5.DWSS, HAdV-C5.IGN, HAdV-C5.TVR, HAdV-C5.KO1, HAdV-C5.KO1 DWSS, HAdV-C5.KO1.IGN or HAdV-C5.KO1.TVR at 5 × 10^3^ vp/cell in the presence of serum-free medium. Cell transduction was measured by luciferase activity 48 h post-infection and normalised for protein content by bicinchoninic acid assay (RLU/mg). Data are represented as the mean RLU/mg protein ± S.D. for each triplicate sample (*n* = 4 experiments). For simplicity, HAdV-C5 is abbreviated to Ad5 in the figure.

We tested the efficiency of our panel of HAdV vectors in transducing CAR^low^/FRα-positive SKOV3 cells. Unfortunately, our data demonstrated that the incorporation of FRα-binding oligopeptides did not significantly enhance transduction via FRα in comparison with the parental HAdV-5 vector (Fig. 5), regardless of the CAR-binding status of the parental virus. Unmodified, control HAdV5 showed luciferase expression of 9.8 × 10^4^ RLU/mg protein, viruses HAdV-C5.DWSS, HAdV-C5.IGN and HAdV-C5.TVR achieved levels of 4.36 × 10^4^, 4.59 × 10^4^ and 4.68 × 10^4^ RLU/mg protein, respectively. Similarly, the HAdV-C5.KO1 control virus showed luciferase expression of 2.87 × 10^4^ RLU/mg protein, whereas viruses HAdV-C5.KO1.DWSS, HAdV-C5.KO1.IGN and HAdV-C5.KO1.TVR achieved levels of 3.95 × 10^4^, 3.1 × 10^4^ and 1.36 × 10^4^ RLU/mg protein, respectively.

### Evaluation of cell binding and trafficking of FRα retargeted adenoviral vectors

**Fig. 6:**
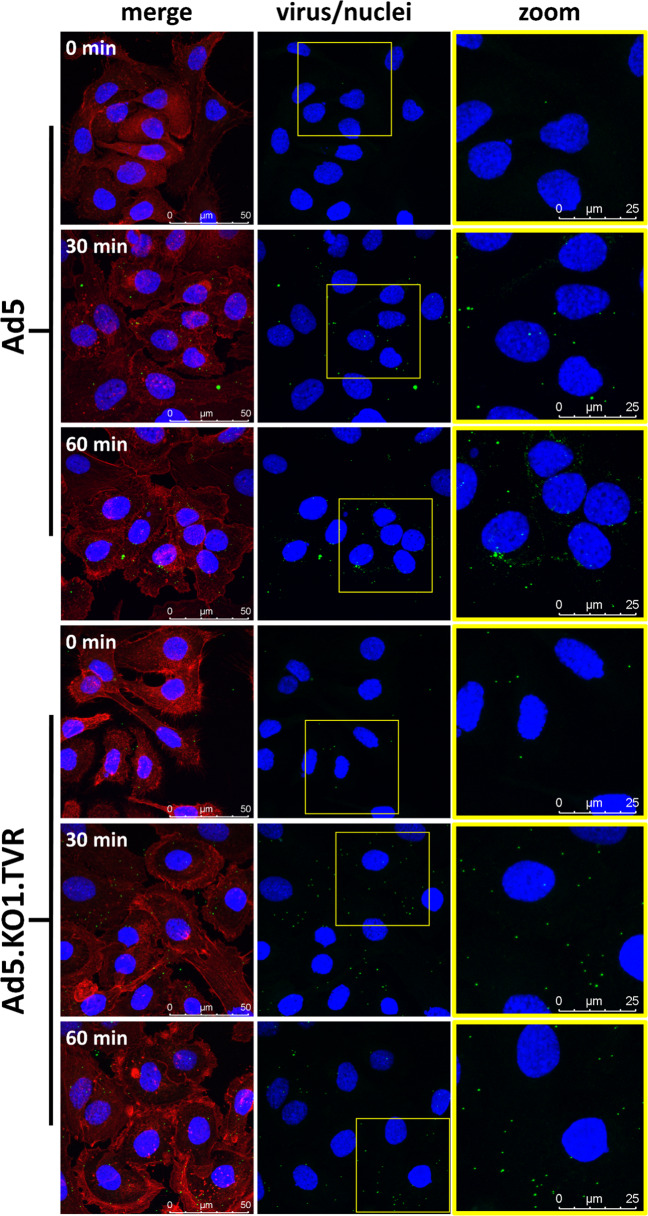
Binding and trafficking of fluorescently labelled HAdV-C5 and HAdV-C5.KO1.TVR in SKOV3 cells. Cells were incubated with 250,000 vp/cell of labelled virus in serum-free medium and incubated on ice for 45 min. Cells were then transferred to 37 °C and imaged after 0, 30 or 60 min. Representative immunofluorescence images are shown. Green; Alexa Fluor 488-labelled HAdV-C5 or HAdV-C5.KO1.TVR; blue—nuclei stained with DAPI; red—actin cytoskeleton stained with Alexa Fluor 555 Phalloidin. For simplicity, HAdV-C5 is abbreviated to Ad5 in the figure.

To assess whether the limited transduction ability observed using FRα retargeted adenoviral vectors was due to defects in cellular binding to FRα or impaired intracellular trafficking pathways of virions post-entry, we performed confocal imaging studies using fluorescently labelled HAdV-C5-based vectors. We restricted this analysis to the purely CAR-engaging HAdV-C5 vectors, and the fully FRα retargeted vector HAdV-C5.KO1.TVR. Confocal imaging of viral attachment and trafficking demonstrated that the FRα retargeted HAdV.KO1.TVR vector can attach and internalise into SKOV-3 cells more efficiently than the CAR-engaging HAdV-C5 vector (Fig. 6). This is likely due to the relatively low expression levels of CAR on SKOV-3 cells (Fig. 5a) compared to high-level expression of FRα (Fig. 1a). Internalised HAdV-C5 was observed to traffic efficiently to the microtubule-organising centre (MTOC), accumulating within the perinuclear region by 60 min. Conversely, FRα internalised HAdV-C5.KO1.TVR appeared to be unable to traffic efficiently to the MTOC or the perinuclear region. Taken together, these data indicate that whilst FRα retargeted viral vectors appear compatible with cell binding and uptake, entry via the FRα is not compatible with efficient viral trafficking post-entry, resulting in inefficient delivery of virions to the nuclear pore complex (NPC).

## Discussion

HAdV-C5 is the most commonly used HAdV vector for gene and virotherapy applications; however, the clinical utility of HAdV-C5 is severely hampered by a lack of tumour specificity, neutralisation by pre-existing antibodies and interactions with blood coagulation factors that sequester adenovirus to the liver [24, 32–40]. To improve the specificity of HAdV vectors for ovarian cancer cells, we developed a panel of vectors retargeted to the FRα by genetically incorporating peptides identified to bind FRα from phage biopanning. Peptides were genetically incorporated in a permissive region of the HAdV-C5 fibre knob HI loop, the capsid protein involved in cell binding. Phage display represents a powerful technique for determining cancer-targeting ligands for various targets in cancer cells [41].

We identified three phage peptides DWSSWVYRDPQT, CIGNSNTLC and CTVRTSAEC that showed binding specificity to SKOV3 cells in vitro. Phage recovery of from SKOV3 cells identified CTVRTSAEC as the lead peptide. We show that synthetic TVRTSAE peptide binds SKOV3 cells in a dose-dependent manner and binds via the FRα since binding was inhibited when cells were pre-treated with the FRα ligand folic acid. In similar studies, peptide MHTAPGWGYRLS was identified as a FRα-specific peptide using the same Ph.D–12 phage-display library for biopanning SKOV3 cells [42]. Peptide SWQIGGN was found to target HO8910 ovarian cancer cells in vitro, inhibiting cell viability, migration invasion and cell adhesion. SWQIGGN peptide treated HO8910 cells injected into BALBC/c nude mice showed reduced volume of ascites and tumour nodule formation in vivo in comparison to control peptide-treated cells [43]. Peptide NPMIRRQ has also been reported as a specific binder to HO8910 ovarian cancer cells in vitro [44] demonstrating plasticity in the sequence of binding peptides for target cells. To the best of our knowledge, our study is the first to report identification of CTVRTSAEC peptide from phage display biopanning. A similar peptide CTVRTSADC differing by a substitution of glutamic to aspartic acid (both charged amino acids) specifically targets prostate cancer cells in vivo [45]. The same Ph.D–C7C phage library as in our study was used for biopanning extradomain-B fibronectin (EDB-fibronectin), a marker of epithelial–mesenchymal transition (EMT) in prostate cancer. Cy5-labelled CTVRTSADC demonstrated increased binding to upregulated EDB-fibronectin in prostate cancer (PC3) cells in vitro and in vivo following intravenous injection in nude mice bearing PC3-GFP tumour xenografts. Interestingly, TVRTSAD and a linear IGNSNTL version of the cyclised CIGNSNTLC peptide discovered herein were reported to target human lung cancer cells in an in vivo mouse model of biopanning [46]. Mice bearing an A549-derived xenograft tumour were intravenously injected with the Ph.D.−C7C phage library into the tail vein. Four rounds of biopanning were performed and phage clones determined from resected tumour. Both TVRTSAD and IGNSNTL peptides were amongst the most commonly occurring phage peptides, however, when peptides were labelled with Cy5.5 and injected into the tail vein of the same mouse model. Although both peptides demonstrated specific targeting to the A549 xenograft tumour, they failed to accumulate in the tumour as abundantly as other candidate peptides and hence were not studied further.

DWSSWVYRDPQT peptide identified in this study reportedly targets colon cancer cells in vitro with in silico analysis suggesting the peptide targets glypican-3 (a heparin sulphate proteoglycan (HSPG)) [47]. We checked all peptides identified in this study with SAROTUP (Scanner and Reporter of Target Un-related peptides). The DWSSWVYRDPQT peptide is considered a potentially false-positive peptide where the sequence WXXW binds plastic with no actual affinity towards the target but rather the (plastic surface) solid phase [48], although data from others [47] would suggest otherwise.

We developed a panel of retargeted HAdV vectors by genetic incorporation of each of the phage peptides into the HI loop of the adenoviral fibre knob, the capsid protein that interacts with the native adenoviral receptor, CAR, and considered an appropriate location for targeting moieties [49, 50]. We evaluated the transduction capabilities of each retargeted HAdV-C5 vector with and without a KO1 mutational background that precludes interactions of adenovirus with the native CAR. Our data show that all retargeted HAdV vectors, regardless of whether CAR binding or not, poorly transduced SKOV3 cells in comparison to the parental HAdV-C5 vector except for HAdV-C5.KO1.DWSS that marginally increased transduction in single experiments (data not shown) but not overall when repeated in a total of four experiments. These data are in concordance with a similar study that genetically incorporated known tumour targeting peptides isolated from in vivo phage-display biopanning; RGD, NGR and ASL into the HI loop of HAdV-5. HAdV vectors retargeted by incorporating peptides in the HI loop and de-targeted with CAR and HSPG mutations showed minimal improvements in transduction of a number of cell types expressing low CAR and in some cases, attenuated adenoviral transduction in comparison to de-targeted vectors with no peptide insertion [51]. Other studies show that incorporation of the RGD motif into the HI loop can reduce native CAR binding [52]. One possible explanation for their findings is the conformational structure of the presented peptide. Transduction of HAdV vectors presenting the cyclised NGR peptide (flanked by cysteine residues and disulphide bond formation confirming a cyclic structure) in the HI loop retargets adenovirus to APN, whereas HAdV vectors containing linear NGR peptide retargets adenovirus to aminopeptidase N (albeit with a lower affinity) [23]. This is one possible explanation as to why elucidated peptides may fail to target efficiently outside of the context of the phage virion from which the phage peptide was selected during biopanning. An alternative explanation is that the presentation of the peptide within the fibre knob protein may be compatible with receptor engagement at the cell surface, but the route of cell entry is not compatible with efficient post-entry trafficking of the internalised virion. Interestingly, our confocal analysis of labelled HAdV-C5 and HAdV-C5.KO1.TVR particles appear to demonstrate that the latter is the case here. FRα retargeted vectors generated in this study were clearly compatible with efficient FRα-mediated cell attachment and internalisation, but were defective in intracellular trafficking post entry, resulting in a poor overall transduction efficiency.

In summary, we demonstrate that TVRTSAE peptide binds FRα on SKOV3 cells in vitro and may represent a potential platform for peptide conjugated drug or gene delivery via liposomes or other viral or non-viral gene transfer applications. In the context of retargeted HAdVs, our panel of peptides failed to enhance FRα-mediated transduction in SKOV3 cells due to defects in intracellular trafficking. Further studies to characterise the reasons for the defective trafficking of FRα-retargeted HAdV-based vectors are warranted.
